# Isolation of Actinomycetes with Cellulolytic and Antimicrobial Activities from Soils Collected from an Urban Green Space in the Philippines

**DOI:** 10.1155/2021/6699430

**Published:** 2021-03-17

**Authors:** Jann Eldy L. Daquioag, Gil M. Penuliar

**Affiliations:** ^1^Institute of Biology, National Science Complex, University of the Philippines Diliman, Quezon City 1101, Philippines; ^2^Natural Sciences and Research Institute, National Science Complex, University of the Philippines Diliman, Quezon City 1101, Philippines

## Abstract

Actinomycetes are one of the most important groups of soil bacteria that are recognized as sources of commercially important enzymes and antimicrobials. Actinomycetes, however, are largely underestimated and uncharacterized in underexplored habitats such as green spaces in urban areas. This study aimed to isolate actinomycetes from soils in the University of the Philippines Diliman campus and determine their cellulolytic and antimicrobial activities. A total of 235 isolates were purified from the soil samples collected. Cellulolytic and antimicrobial activities were observed in 114 and 18 isolates, respectively. The cell-free supernatants of 31 isolates exhibited high cellulolytic activities. Two isolates, in particular EWLG2 and EPNA9, had 0.596 FPU and 0.885 FPU cellulolytic activity, respectively. Seven isolates exhibited antimicrobial activities in the screening methods used. The crude extracts of isolates AWLG9, AWLG8, AWLG10, AULG1, APLG2, and AWLG13 had minimum inhibitory concentrations (MIC) values ranging from 31.25 *µ*g/mL to 500 *µ*g/mL against *Candida* spp. Isolates AULG1 and EPLG5 were active against the bacterial test microorganisms and had MIC values ranging from 250 *µ*g/mL to 500 *µ*g/mL. DNA sequencing identified the isolates which exhibited high cellulolytic and antimicrobial activities as *Bacillus* sp. and *Streptomyces* sp., with percent identities ≥98%. This study shows that green spaces are rich sources of soil microorganisms that have cellulolytic and antimicrobial activities. It is recommended that the isolates obtained in this study be examined further for possible applications in bioethanol production and pharmacology.

## 1. Introduction

Microorganisms have long been shown to be sources of natural products with industrial and medical applications. Soil microorganisms, in particular, have been widely studied for their ability to produce diverse compounds such as enzymes and biologically active secondary metabolites [1, 2]. One of the most important groups of soil bacteria, recognized as a source of commercially important enzymes and antimicrobials, is the actinomycetes.

Actinomycetes are Gram-positive, generally spore forming bacteria belonging to class Actinobacteria and order Actinomycetales. They are taxonomically known for their high guanine to cytosine (G-C) content of over 55%, and their fungal-like colony morphology [3, 4]. The most common genera of actinomycetes are *Actinomyces, Actinoplanes, Micrococcus,* and *Streptomyces* [5, 6].

Actinomycetes are widely distributed in nature and have been isolated from aquatic and terrestrial environments. They are part of the normal microflora of the soil and constitute a significant component of the soil microbial community. They are known to be responsible for the production of about half of the discovered biologically active secondary metabolites with antimicrobial activities [7]. They contribute to the degradation of soil organic material by producing enzymes that can degrade cellulose, hemicellulose, and lignin [1, 2, 8]. Cellulose is the most abundant organic compound on Earth and is the major waste generated by the agricultural industry, which can be utilized as substrates for industrial purposes [1, 9]. The agricultural wastes of the Philippines are said to be the most underdeveloped biomass resource for sustainable bioenergy; however, these agricultural wastes currently have no established commercial value [10].

The diversity of actinomycetes is largely underestimated in unexplored and underexplored habitats. This, combined with their ability to produce unique enzymes and secondary metabolites, results in the great interest in isolating novel species of actinomycetes. Many of these yet to be discovered actinomycete species may have the potential to produce enzymes and antimicrobials with industrial and medical applications [11, 12].

In the Philippines, no significant studies have been conducted to isolate and characterize actinomycetes with enzymatic and antimicrobial activities from green spaces located in cities.

Urbanization in cities has detrimental effects on the soil ecosystem through pollution discharge, human disturbance, and changes in urban climate. These lead to changes in the soil properties resulting in changes in soil bacterial community [13]. Urban factors including population density and green spaces type lead to significant differences in the microbial community composition [14]. The University of the Philippines (UP) Diliman is one of the last green spaces in Metro Manila [15] and may likely support diverse communities of actinomycetes. The ecological pressure brought by urbanization around the University and the disturbances brought by visitors through the years may have selected for actinomycetes with novel activities.

Considering the potential of discovering actinomycetes that can produce enzymes and antimicrobials with industrial and medical applications, this study aimed to isolate actinomycetes from soil samples collected from selected sites in UP Diliman, to screen the isolates for cellulolytic and antimicrobial activities, and to identify isolates that exhibit the highest cellulolytic and/or antimicrobial activities by morphological, molecular, and biochemical methods. This study was limited to actinomycete isolation from soil samples collected inside the campus with antimicrobial activities against selected pathogens, and those with cellulolytic activities.

## 2. Materials and Methods

### 2.1. Site Description

Four locations served as sampling sites for this study: the Lagoon, New Arboretum, Sunken Garden, and Old Arboretum of the University of the Philippines Diliman. The Lagoon is a small body of water more than a meter deep that is surrounded by various species of trees such as *narra*, *banaba*, and *kapok.* It is perpetually covered with algal growth and supports various fish species including mudfish, catfish, and tilapia. The New Arboretum is a 1-hectare plot of land with more than 150 trees representing 79 endangered species while the Old Arboretum is a 16-hectare plot of disturbed habitat that was originally a grassland that eventually evolved into a man-made forest. The Sunken Garden, on the other hand, is a 5-hectare natural depression of land that is almost perpetually covered with grass. It is used for various recreational activities by students and residents of the campus.

### 2.2. Sample Collection and Treatment

One hundred grams of soils (wet weight) were collected 5 to 20 cm below the surface of decaying leaf litters in each site. The soils were air-dried at room temperature for 10 d and were then passed through a 2- mm sieve before undergoing different treatments to facilitate the isolation of different microorganisms [16, 17]. The treatments used were phenol exposure (1.5% v/v) at 30°C for 30 min and wet heat exposure at 65°C for 30 min [18].

### 2.3. Isolation and Purification

The treated soil samples were suspended in 0.9% sterile saline solution and serially diluted until 10^−5^ [19]. Aliquots of 100 *µ*L from the last three dilutions were plated on three isolation media supplemented with nystatin (50 mg/L) as antifungal agent [20]. The media used were actinomycete isolation agar (AIA), Emerson's agar (EA), and soybean casein digest agar (SCDA) [21–23]. The plates were incubated at 28°C for up to 14 d to isolate slow growing actinomycetes. Plates were observed daily for bacterial colonies exhibiting typical features of actinomycetes. Colonies that appeared dry, with or without pigments, and with aerial or substrate mycelium were considered as putative actinomycetes [24]. Selected colonies were purified by three-way streak method and were maintained in agar slants of their respective isolation medium [25].

### 2.4. Determination of Cellulolytic Activity

Isolates were screened qualitatively for cellulolytic activity by observation of a clearance zone around their growth on carboxyl methyl cellulose (CMC) agar . An agar plug (5 mm diameter) of the isolate grown for 7 d on their respective isolation medium was imprinted on CMC agar plates and was incubated at 28°C for 48 h. The cellulolytic activity was visualized by flooding the plates with Gram's iodine for 3 to 5 min, and the diameter of the clearance zones was measured using a Vernier caliper [26, 27]. This assay was carried out twice with three replicates each trial. Isolates with clearance zones of at least 20 mm were selected for the filter paper activity (FPase) assay [28]. Cell-free supernatants (CFS) of the isolates were obtained by submerged state fermentation, where standardized suspension of the isolates was inoculated into 100 mL CMC broth supplemented with glucose (0.2% v/v) and incubated at 28°C for 7 d at 200 RPM. The CFS were collected by centrifugation at 10,000 RPM for 10 min and used for secondary screening [29]. Briefly, 0.5 mL of CFS of each isolate was added to a Whatman No. 1 filter paper strip (1 cm × 6 cm, 50 mg) immersed in 1 mL of 0.05 M sodium citrate buffer (pH 4.8). The reactions were incubated at 50°C for 60 min and terminated by adding 3 mL 3,5-dinitrosalicylic acid (DNS) reagent. The color of the reactions was developed by incubating them at 100°C for 5 min, followed by incubation in a cold-water bath. Twenty milliliters deionized water was added to each mixture and left to stand for 20 min before reading the absorbance at 540 nm [28, 30]. A solution of the reagents without the CFS was used as a spectro-zero, while a solution of the reagents without the cellulose source was used as an enzyme blank. A standard curve with glucose as carbon source was constructed, and the amount of glucose detected in the FPase assay was calculated using [30]

(1) $$\begin{array}{c} \text{FPU}=\frac{\left(0.37/\left(\text{enzyme concentration to release }2.0\text{ mg glucose}\right)\right)\text{units}}{\text{mL}}. \end{array}$$

One unit of filter paper (FPU) is defined as the amount of substrate converted to glucose by the enzyme in *µ*M per min averaged over 60 min [28, 30]. This assay was carried out twice with two replicates each trial. Isolates with the highest FPU were identified using morphological, biochemical, and molecular methods.

## 3. Determination of Antimicrobial Activity

### 3.1. Test Organisms

The test microorganisms used in antimicrobial screening were *Pseudomonas aeruginosa* BIOTECH 1335, *Klebsiella pneumoniae* BIOTECH 1754, *Salmonella enterica* serovar Typhi BIOTECH 1756, *Escherichia coli* BIOTECH 1634, *Enterococcus faecalis* BIOTECH 10348, *Staphylococcus aureus* BIOTECH 1350, Methicillin-resistant *S. aureus* BIOTECH 10378, *Bacillus subtilis* BIOTECH 1679, *Candida albicans* MML JPCA1301+, *Candida tropicalis* MML JPCT1301+, *Saccharomyces cerevisiae* BIOTECH 2030, *Fusarium oxysporum* BIOTECH 3429, and *Aspergillus flavus* BIOTECH 3092.

### 3.2. Screening for Antimicrobial Activity

Primary and secondary screening were used to identify isolates with antimicrobial activity. In the primary screening, agar plug diffusion assay was performed [31, 32]. Briefly, agar plugs of the isolates grown in their respective isolation medium were made on the 7^th^ and 14^th^ d of incubation and were screened against standardized lawns of the test microorganisms on Mueller Hinton Agar (MHA) plates. After incubation at 37°C for 24 h, the diameters of the zones of inhibitions (ZOI) were measured [32]. Isolates with an activity in at least one test microorganism were selected for secondary screening [31]. In the secondary screening, agar well diffusion assay was performed. Briefly, CFS generated by submerged state fermentation using yeast malt broth (YMB) were used. The pH of the CFS were measured before the assay and 50 *µ*L CFS were placed in wells bored on MHA plates seeded with the test microorganisms. After incubation at 37°C for 24 h, the ZOI were measured using a Vernier caliper [31–33]. These assays were carried out in triplicate. Isolates with the highest ZOI were selected for optimization of their antimicrobial activity [29, 33].

### 3.3. Optimization of Antimicrobial Activity

Minimal salt medium (M9), M9 supplemented with 2% glucose, and YMB were used to optimize the antimicrobial activity of the selected isolates. The media were seeded with standardized inocula of the isolates and incubated at 28°C for 14 d at 200 RPM. CFS were collected every 24 h and tested for antimicrobial activity using the agar well diffusion assay [29, 33]. The agar well diffusion assay was carried out in triplicate. The medium and the incubation time which resulted in the largest ZOI were noted and used for the extraction of the biologically active compounds [34].

### 3.4. Extraction of Biologically Active Compounds

Production of biologically active compounds was done by submerged state fermentation using the media and incubation time that produced the highest antimicrobial activity. Briefly, 2.5 L of YMB was seeded with standardized inoculum of the isolate and incubated for 6 d at 28°C at 200 RPM. After fermentation, CFS was collected by centrifugation at 10,000 RPM for 10 min. The CFS was mixed with equal volume of ethyl acetate, shaken vigorously, and incubated at room temperature for 60 min. The solvent phase was collected using a separatory funnel and concentrated using a rotary evaporator at 40°C under reduced pressure. The extracts were dried and dissolved in 100% DMSO to prepare a stock concentration of 30 mg/mL [19, 34].

### 3.5. Determination of Minimum Inhibitory Concentration

The minimum inhibitory concentration (MIC) of the ethyl acetate crude extract of the isolates was determined using broth microdilution method [33]. The crude extracts were first diluted using sterilized deionized distilled water to reach a solvent concentration of 10% DMSO, after which they were two-fold serially diluted to produce concentrations of 1000 *µ*g/mL to 1.95 *µ*g/mL. One hundred microliters of each concentration were added to 100* µ*L of 2X MHB inoculated with standardized suspension of the test microorganisms to reach final crude extract concentrations ranging from 500 µg/mL to 0.98 µg/mL. This was followed by incubation at 37°C for 24 h [33, 35]. This assay was carried out twice with three replicates per trial. The MIC was then determined as the lowest concentration of crude extract that inhibited the growth of the test microorganism.

## 4. Identification of Isolates

### 4.1. Gram Staining

A loopful of the isolate was smeared on a glass slide, air-dried, and heat-fixed. The smear was covered with crystal violet for 30 s and washed off with distilled water. Gram's iodine solution was added and left to stand for 1 min and then washed off with distilled water. The smear was decolorized with 95% ethanol dropwise for 15 to 20 s and washed off with distilled water. The smear was flooded with safranin for 1 min, washed with distilled water, and dried. The smear was examined under a bright field microscope using 1000*x* magnification [36, 37].

### 4.2. Morphological Characterization

Isolates were streaked onto their respective isolation and incubated at 28°C for 7 d. Colony morphology was noted with respect to pigment production, absence or presence of aerial and substrate mycelium, and nature of colony [38, 39].

### 4.3. Biochemical Characterization

Gram-positive isolates were selected for biochemical characterization. The biochemical tests performed were catalase test, citrate utilization, urea hydrolysis, indole test, methyl red test, and Voges-Proskauer test [40].

## 5. Molecular Identification

The DNA of the selected isolates was extracted based on the methods of Valli and colleagues with some modifications. Universal primers 27F (5′-GAGTTTGATCCTGGCTCA-3′) and 1492R (5′- TACGGCTACCTTGTTACGACTT-3′) were used for PCR amplification, using conditions optimized in the laboratory [41–43]. PCR amplification was carried out in 25 *µ*L reactions containing 12.5 *µ*L of GoTaq® Green Master Mix (Promega Madison, WI, USA), 8.5 *µ*L of sterile distilled water, 1.5 *µ*L (0.6 *µ*M) of each primer, and 1 *µ*L (>100 ng/*µ*L) of template DNA. PCR conditions used were initial denaturation at 94°C for 5 min, 30 cycles at 94°C for 45 s, annealing at 42°C to 72°C for 1 min and elongation at 72°C for 40 s, and final extension at 72°C for 10 min [43]. PCR products were electrophoresed in 1% agarose gels and visualized under a UV transilluminator [44]. PCR products of the universal primers were sent to Macrogen (Korea) for sequencing, and the sequences were processed and analyzed using the Staden Package v2.0.0 and BioEdit v7.2.5 program [45]. The consensus sequences were compared to reference sequences of actinomycetes and other bacterial sequences using the BLASTN program of the National Center for Biotechnology Information (http://www.ncbi.nlm.nih.gov). Isolates were identified based on a sequence similarity of ≥98.7% [46].

### 5.1. Phylogenetic Analysis

GenBank full formats of the partial 16S rRNA sequences of 23 *Bacillus*-type strains and 48 *Streptomyces*-type strains were downloaded from the nucleotide database of National Center for Biotechnology Information (NCBI). Eight sequences of bacteria belonging to the genera *Paenibacillus, Brevibacillus,* and *Alicyclobacillus* were also downloaded and used as the outgroup taxa for the *Bacillus* phylogenetic analysis. Seven sequences of bacteria belonging to *Nocardia, Kitasatospora,* and *Corynebacterium* were also downloaded and used as the outgroup taxa for the *Streptomyces* phylogenetic analysis. The downloaded sequences along with the sequences of the isolates were aligned and trimmed using ClustalW in the BioEdit v7.2.5 program [45]. The consensus sequences had a total length of 1331 nucleotides and 1337 nucleotides for *Bacillus* and *Streptomyces,* respectively. The sequences were then analyzed for optimal nucleotide substitution model testing using jModelTest 0.1.1 [47]. There was a total of 88 different models of nucleotide substitution using the Akaike Information Criterion (AIC) of model selection and the models with the lowest AIC score, GTR + I + G for *Bacillus* and TIM1+I + G for *Streptomyces*, were selected. The sequences were then evaluated for oversaturation using the Xia Test in DAMBE [48, 49]. The oversaturation of the datasets was determined by comparing the value of the simple index of substation of saturation (Iss) to the critical values for the dataset based on completely symmetrical and extremely asymmetrical tree. The construction of phylogenetic trees was performed using neighbor-joining (NJ) method in PAUP^∗^ version 4.0b10 [50], with 1000 non-parametric bootstraps used as replicates. The generated tree was rendered using TreeExplorer version 2.12 [50].

## 6. Results and Discussion

Actinomycetes have been extensively studied in different environments and habitats in the last decades. However, there is little to no report regarding the isolation of actinomycetes from green spaces in urban places. This study aimed to isolate actinomycetes from this unexplored habitat to find species with cellulolytic and antimicrobial activities.

**Figure 1:**
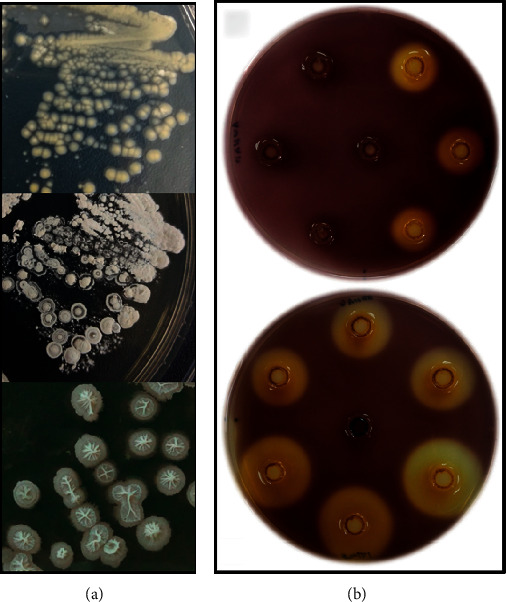
Representative colony morphology of isolated colonies and plates showing cellulolytic activity of isolates. (a) Bacterial isolates showing the typical characteristic of actinomycetes. (b) Representative plates of the cellulolytic activity of selected isolates in CMC agar flooded with Gram's iodine showing isolates with clearance zone diameters of at least 20 mm and with clearance zone diameters lower than 20 mm. The middle imprints on both plates are negative controls showing no clearance zones.

A total of 385 distinct colonies were isolated from the soil samples, based on the typical colony features of actinomycetes (Figure 1). Only 235 isolates, however, were successfully purified. Primary screening of the isolates showed that 48.51% (114/235) of the isolates had cellulolytic activity. The highest number of isolates that exhibited cellulolytic activity were collected from the Lagoon (28.95%, 33/114), followed by the New Arboretum (26.32%, 30/114), Sunken Garden (23.68%, 27/114), and Old Arboretum (21.05%, 24/114). The differences in the number of isolates with cellulolytic activity between sites were not significantly different. This may be due to the high microbial load of soils and ubiquity of cellulose-degrading microorganisms in soils [8]. Out of the 114 isolates, only 31 (27.19%) had clearance zone diameters of at least 20 mm (Figure 1).

The clearance zones demonstrate the ability of the isolates to utilize the cellulose present in the medium, and the differences in the cellulolytic activities of the isolates are likely due to differences in the vegetation litter and the microclimate of the sampling sites [26]. All sampling areas were covered with different vegetation and different microclimates are observed in the sampling sites. This may have affected the input of abiotic and biotic factors that may have favoured the presence and the activities of cellulose-degrading microorganisms [51]. The difference in the cellulose degradation of microorganisms under different vegetation types and microclimates was also observed by Chen and colleagues. They observed that vegetation, litter types, and microclimatic factors have effects in the ligninolytic and cellulolytic enzyme activities [52]. Isolates with clearance zone diameters of at least 20 mm were selected for enzyme production by submerged state fermentation for the quantitative screening of cellulolytic activity.

In the FPase assay, only two isolates, EWLG2 and EPNA9, showed high cellulolytic activities with 0.596 FPU and 0.885 FPU, respectively. The other isolates had low to no cellulolytic activity. This finding agrees with the results of Teather and Wood when they observed that there was no apparent correspondence between the diameters of the clearance zones on CMC agar and the rate of insoluble cellulose degradation [53]. These results are also comparable to those reported by Liang and colleagues where most of their isolates with clearance zones in CMC agar had undetectable FPase activity [54].

The loss of the cellulolytic activity of the isolates on the FPase assay may be due to the low concentration of enzymes present in the supernatants used,resulting to it being not detected. It could also be due to the type of cellulase that the isolates produced, which may be intracellular or extracellular. Isolates that had no to low cellulolytic activity may have produced cellulases that were cell associated, which are not secreted in their supernatants. Finally, the conditions used in the production of the supernatants may not be optimal for the secretion of cellulases in most of the isolates [1]. The two isolates with cellulolytic activity were then morphologically and biochemically characterized (Table 1). The two isolates, EWLG2 and EPNA9, were molecularly identified as *Bacillus cereus* (99.86%) and *B. pseudomycoides* (99.29%), respectively (Table 2).

The isolation of *Bacillus* when targeting actinomycetes from soils was also observed in the study conducted by Duraipandiyan and colleagues wherein their isolates were identified as *Bacillus* even when they were selectively isolating for actinomycete [55]. This may be due to the similar colony morphology of some actinomycetes and *Bacillus,* combined with the wide range of morphologies the colonies of *Bacillus* present. This was also observed in the study of Eppard and colleagues where they isolated actinomycetes from soils and one of their isolates turned out to be*Bacillus.* The colony morphology of this isolate varied considerably depending on the media used, presenting colony morphology characteristics of actinomycetes until it was further identified using 16S rRNA sequencing [56].

The cellulolytic activity of *B. cereus* reported in this study is higher compared to those observed by Chantarasiri and colleagues. Their study showed that the supernatants collected under submerged state fermentation of their isolated *B. cereus* had cellulolytic activity of 0.057 FPU [57]. While the cellulolytic activity of the isolates from this study was lower compared to the 3.69 FPU reported by Lah and colleagues, it should be noted that they used palm kernel cake as the substrate. The higher cellulolytic activity of *B. cereus* in their study was likely due to the different method used in producing the CFS. The CFS used in their study was more concentrated, compared to the CFS used in this study, resulting in a higher cellulolytic activity [58]. The conditions for the enzyme production of the isolates were also not optimized in this study, while the pH, incubation time and temperature, and media composition were optimized in their study [57, 58].

The cellulolytic activity of our isolates is also higher than those reported in other studies on the cellulolytic activity of other microorganisms. The cellulolytic activity of the crude extracts of *Anoxybacillus* in the work of Liang and colleagues ranged from 0.1 to 0.7 FPU [59], while the cellulolytic activity of eight bacterial isolates reported by Gupta and colleagues showed a range of 0.012 to 0.196 FPU [60]. They also showed the ability of their isolates co-cultured with *Saccharomyces cerevisiae* in bioethanol production by simultaneous saccharification and fermentation. They reported fermentation and ethanol production by the synergistic cellulose degradation and fermentation of degraded cellulose that is converted to ethanol [60]. The isolates in this study had higher cellulolytic activities than those of Gupta and colleagues and can be further studied for their cellulolytic potential for actual application in the conversion of waste products into value-adding and useful products. The *Bacillus* isolates in this study had moderate cellulolytic activity compared to these studies on *Bacillus* and other bacteria with cellulolytic activity [57, 58, 61].

Out of the 235 isolates, 52 isolates remained viable after repeated subculture, and only 34.62% (18/52) showed antimicrobial activity in the primary screening against at least one of the test microorganisms used. Ten isolates showed antibacterial activity against *S. mutans, B. subtilis, P. aeruginosa,* and *S. enterica*serovar Typhi, while 14 isolates inhibited the growth of *S. cerevisiae, C. albicans,* and *C. tropicalis* (Table 3). Five isolates showed antifungal activity against *F. oxysporum* and *A. flavus.*

**Figure 2:**
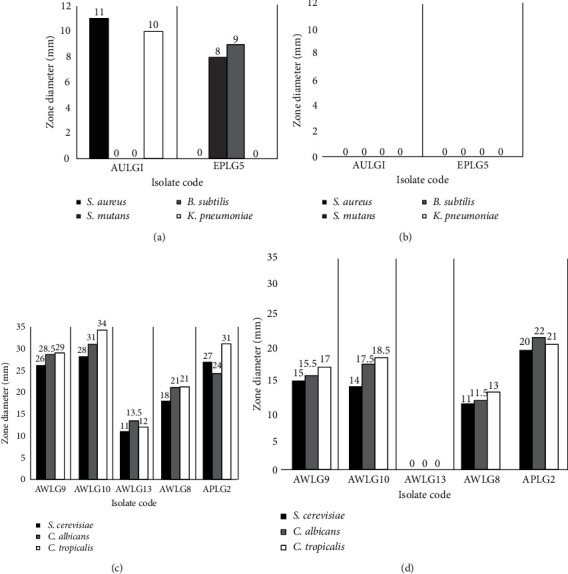
Antimicrobial activity of the isolates against the selected test microorganisms using agar well assay. Zone of inhibition diameter of the supernatants of the isolates against bacterial test microorganisms on the (a) 7^th^ and (b) 14^th^ day of incubation, and against yeast test microorganisms on the (c) 7^th^ and (d) 14^th^ day of incubation.

Out of all the isolates with activity on the primary screening, only 38.89% (7/18) showed antimicrobial activity against at least one of the test microorganisms in the secondary screening. The supernatants were collected on the 7^th^ and 14^th^ d of incubation in submerged state fermentation. Supernatants collected on the 14^th^ d of incubation did not retain their antibacterial activity, while four isolates retained their antifungal activity against the yeasts used (Figure 2). It was observed that the supernatants collected on the 14^th^ d had lower zones of inhibition compared to supernatants collected on the 7^th^ d of incubation.

The agar plug diffusion assay was used as a primary screening method to determine the range of the inhibitory activity of the isolates against various test microorganisms [4], while the agar well diffusion assay was used as a secondary screening method to determine if the isolates will retain their inhibitory activity even without cell-to-cell contact with the test microorganisms. If the inhibitory activity of the isolates was retained in the agar well diffusion assay, it may mean that the biologically active compound of the isolate may be extracellular in nature and can be extracted and concentrated to determine its MIC. Before the MIC was determined, the fermentation media and incubation time were first optimized.

All media used to optimize antimicrobial activity supported the growth of all the isolates, as evidenced by the increased turbidity of the media, but only supernatants collected from YMB showed activity against at least one of the test microorganisms. The supernatants collected from the supplemented and unsupplemented minimal media showed no inhibitory activity. Media optimization is a crucial factor for production of biologically active compounds, as proper nutritional composition leads to the expression of genes and triggers the required metabolic pathways [62, 63]. Minimal media with its inorganic nitrogen sources were used to trigger an optimum level of stress condition for the isolates to promote the production of biologically active compounds. The stressful condition may not be suitable for biomass production but is theoretically useful for secondary metabolite production [62, 63]. However, this was not observed in the study. Instead, the complex media used resulted in a higher inhibitory activity. The optimal stress condition for the isolates in this study may be further studied to stimulate the expression of genes encoding for secondary metabolite production. While all media supported the growth of the isolates, it was evident that YMB was most suitable in the production of biologically active metabolites of the isolates.

The largest zones of inhibition were observed from supernatants collected after 5 to 6 d of incubation (see Figures S1–S14 in the Supplementary Materials for the results of the optimization of media and incubation time). It should be noted, however, that antimicrobial activity of the CFS was reduced considerably after further incubation time, which might be due to the degradation of the active compound in the supernatants [19]. The optimized conditions were used for the extraction and concentration of the biologically active compounds of the isolates.

The crude extracts of the isolates were used to determine their MIC against the test microorganisms. Isolates AWLG9, AWLG8, AWLG10, AULG1, APLG2, and AWLG13 were highly active against the yeast test microorganisms, while AULG1 and EPLG5 were active only against some of the bacterial test microorganisms (Table 4). The MIC of the isolates ranged from 31.25–500 *µ*g/mL. These MICs were higher than those of the controls used, indicating that the crude extracts were less effective in inhibiting the growth of the test microorganisms. It should be noted, however, that the crude extracts of the isolates were not pure and concentrated, and some inhibitors might be present in the crude extracts. The crude extracts may be further studied for purification and identification of the active compound. Further characterization of the active compound found in the crude extract of the isolates may lead to a higher antimicrobial activity and lower MICs.

**Figure 3:**
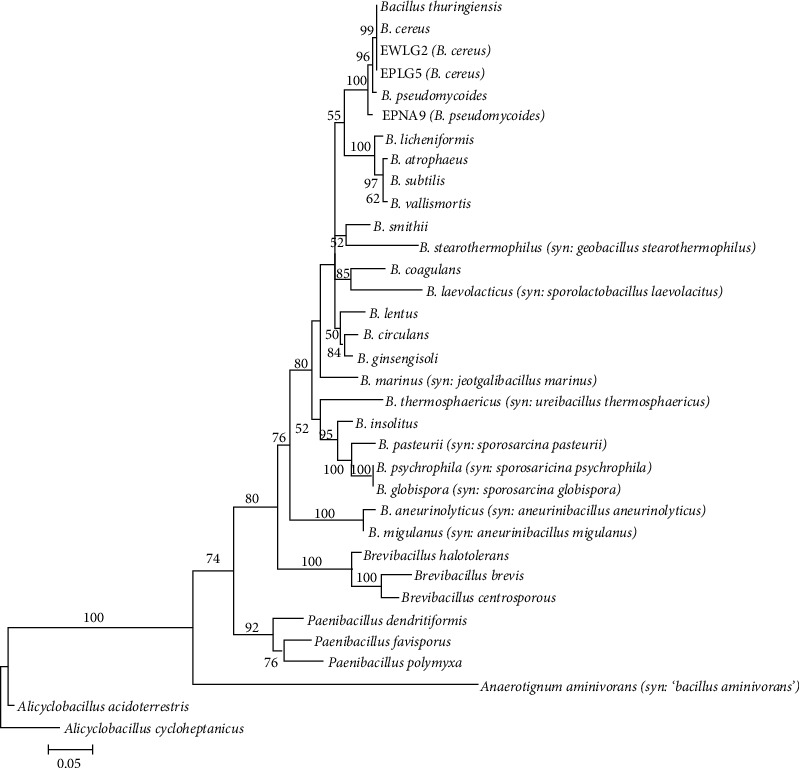
Neighbor-joining tree of the genus *Bacillus* based on 1331 nucleotides of the *16s rRNA* gene using the GTR + I + G model of DNA substitution. The tree is rooted on members of *Paenibacillus, Brevibacillus,* and *Alicyclobacillus.* Values on nodes represent percentage bootstraps out of 1000 bootstrap samples; values less than 50% are not shown. Scale bar represents five nucleotide substitutions for every one thousand nucleotides. The phylogenetic tree supports the BLAST identity of the isolates as they cluster with the *Bacillus cereus*- and *Bacillus pseudomycoides*-type strains.

**Figure 4:**
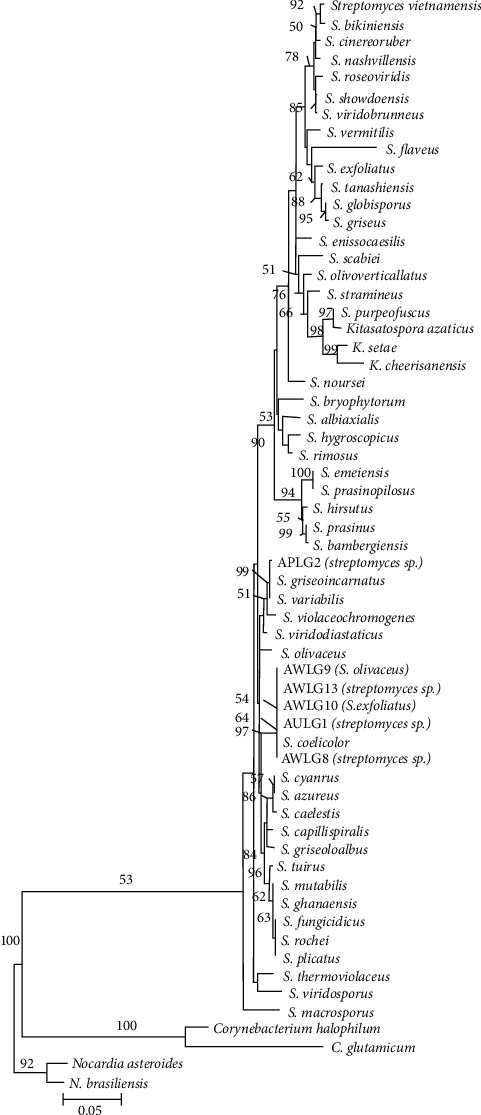
Neighbor-joining tree of the genus *Streptomyces* based on 1337 nucleotides of the *16s rRNA* gene using the TIM1+I + G model of DNA substitution. The tree is rooted on members of *Nocardia, Kitasatospora,* and *Corynebacterium.* Values on nodes represent percentage bootstraps out of 1000 bootstrap samples; values less than 50% are not shown. Scale bar represents five nucleotide substitutions for every one thousand nucleotides.

The 16S rRNA sequences of the isolates were compared to reference sequences in GenBank and revealed that the isolates belong to the genera *Bacillus* and *Streptomyces* with percent identity of ≥98.7% (Table 2). In the phylogenetic trees constructed using the sequences of the isolates and downloaded sequences of various type strains, all the isolates clustered with their closest match based on their BLAST results, with 54–100% NJ bootstrap supports (Figures 3 and 4).

The isolates that exhibited high inhibitory activities against yeast microorganisms were identified as *Streptomyces* (Table 1). *Streptomyces* have been widely studied for their production of medically important secondary metabolites. The results of this study support other studies showing the antifungal properties of *Streptomyces* [64, 65]. The inhibitory activity of *Streptomyces* to fungi is generally related to extracellular hydrolytic enzymes they produce [66]. In this study, the crude extracts of the isolates had lower MIC than nystatin. However, these extracts can be promising for their similarity to nystatin and amphotericin B. These two common antifungals were developed from metabolites produced by *Streptomyces* that were isolated from soils. Nystatin was developed from *Streptomyces noursei* which showed antimicrobial activity against *C. albicans* and *Cryptococcus neoformans,* while Amphotericin B was isolated from *Streptomyces nodosus* and its antimicrobial activity was shown using supernatants collected after fermentation [67–69]. The biologically active compounds obtained in this study may provide novel antifungal compounds if further purified.

Among the different general of actinomycetes, *Streptomyces* is the most recognized genus and is widely distributed in soils [70]. About half of the biologically active compounds sourced from microorganisms are estimated to come from actinomycetes, and the majority of these compounds are derived from *Streptomyces* [71–73]. The secondary metabolites of actinomycetes are potential sources of various novel compounds that have biologically active properties. In this study, some of the isolates exhibited consistent antimicrobial activities from the primary screening to MIC determination. Isolates AWLG9, AWLG8, AWLG10, AWLG13, and AULG1 are promising isolates with not only antimicrobial properties, but also cellulolytic activity shown on the primary screening using CMC agar. Furthermore, isolates AWLG8, AWLG13, APLG2, and AULG1 were identified only in the genus level. These isolates can be characterized further to determine if they are novel species of the genus *Streptomyces.* Although 16S rRNA gene sequencing is used for species-level identification, the identification and taxonomy of *Streptomyces* have been based primarily on morphological, biochemical, and physiological characteristics [74]. Morphological, biochemical, and physiological characteristics, such as utilization of different carbon sources and degradation of amino acids, production of melanin, nutritional requirements, cell wall, and isoprenoid quinones composition, must be studied in order to confidently say that the promising isolates in this study are novel species [74–76]. Furthermore, the crude extracts of the isolates may be purified and characterized to identify the biologically active compounds they produce. These purified active compounds may have a higher antibacterial activity against *B. subtilis, K. pneumoniae, S. aureus, S. mutans,* and other pathogens.

## 7. Conclusions

The results presented in this work show actinomycetes and *Bacillus* isolated from urban green spaces in the Philippines have cellulolytic and antimicrobial activities that have potential industrial and medical applications. The isolates in this study show moderately high cellulolytic and antimicrobial activities. The large number of isolates that had cellulolytic activity on the primary screening may also be greatly underestimated and the ecological pressure brought by urbanization in the sampling sites may be revisited to understand its effect on the distribution and diversity of these industrially important microorganisms. The isolation of seven *Streptomyces* spp. with high antimicrobial activities is also an indication that research on soil microorganisms for medically important secondary metabolites remains a promising endeavour in discovering new antimicrobials.

## Figures and Tables

**Table 1:** Morphological and biochemical phenotypic characterization of isolates with high cellulolytic and antimicrobial activities.

	EWLG2	AWLG9	AWLG8	AWLG10	AWLG13	APLG2	AULG1	EPLG5	EPNA9
Gram-reaction	Positive rod	Positive rod	Positive rod	Positive rod	Positive rod	Positive rod	Positive rod	Positive rod	Positive rod
Catalase test	(+)	(+)	(−)	(+)	(+)	(+)	(+)	(+)	(+)
Indole test	(−)	(−)	(−)	(+)	(−)	(+)	(+)	(−)	(−)
Methyl-red test	(−)	(+)	(−)	(−)	(+)	(+)	(+)	(−)	(−)
Voges-Proskauer test	(+)	(−)	(−)	(−)	(−)	(+)	(+)	(+)	(+)
Urease test	(+)	(−)	(−)	(−)	(−)	(−)	(+)	(+)	(+)
Citrate utilization	(+)	(−)	(+)	(−)	(−)	(−)	(−)	(+)	(+)

**Table 2:** Molecular identification of the isolates with cellulolytic and/or antimicrobial activity using 16S rRNA sequences.

Activity	Isolate	16S rRNA identification	Query cover (%)	Percent identities (%)
Cellulolytic activity	EWLG2	*Bacillus cereus*	100	99.86
	EPNA9	*Bacillus pseudomycoides*	100	99.29

Antimicrobial activity	AWLG9	*Streptomyces olivaceus*	100	98.89
	AWLG8	*Streptomyces* sp.	100	100.00
	AWLG10	*Streptomyces exfoliatus*	100	99.58
	AWLG13	*Streptomyces* sp.	100	99.86
	APLG2	*Streptomyces* sp.	100	99.92
	AULG1	*Streptomyces* sp.	100	99.50
	EPLG5	*Bacillus cereus*	100	99.86

**Table 3:** Antimicrobial activity of the isolates against the selected test microorganisms using agar plug diffusion assay. (A) Zone of inhibition diameter of the isolates against bacterial test microorganisms and yeast test microorganisms.

Zone of inhibition diameters (mm)								
Isolate code	Bacterial test microorganisms					Yeast test microorganisms		
	*B. subtilis*	*E. faecalis*	*P. aeruginosa*	*S. enterica/serovar* Typhi	*S. mutans*	*C. albicans*	*C. tropicalis*	*S. cerevisiae*
AUNA1	20	0	13.5		12	10.5	10.5	14
AUOA 19	17.5	0	0	11.5	7	13	10.5	15.5
EPLG 5	11.5	0	0	0	18	14	13	12
SPLG11	0	17.5	0	0	0	13.5	13	18.5
APNA 10	0	0	18	0	0	10	10.5	10.5
AWLG9	0	0	14	0	0	21	20.5	21
AUOA15	0	0	14	0	0	0	0	0
APLG2	0	0	13.5	0	0	0	0	0
SPOA10	0	0	0	0	23	19	17.5	17
SUOA4	0	0	0	0	16	0	0	0
AWOA19	0	0	0	0	11.5	0	0	0
AWLG10	0	0	0	0	0	17	16	17
AWLG13	0	0	0	0	0	16	14.5	12
SPOA1	0	0	0	0	0	15.5	14.5	16.5
SPSK12	0	0	0	0	0	13	13.5	16
AULG1	0	0	0	0	0	12.5	11.5	12
AWLG14	0	0	0	0	0	10.5	10	13.5
AWLG 8	0	0	0	0	0	8.5	8	8.5

**Table 4:** Minimum inhibitory concentrations of the highly active isolates against the test microorganisms.

Minimum inhibitory concentration (*µ*g/mL)							
	*C. albicans*	*C. tropicalis*	*S. cerevisiae*	*B. subtilis*	*K. pneumoniae*	*S. aureus*	*S. mutans*
AWLG9	125	125	125	—	—	—	—
AWLG8	125	62.5	250	—	—	—	—
AWLG10	250	31.25	250	—	—	—	250
AULG1	125	62.5	125	—	500	250	—
APLG2	—	—	—	—	—	—	—
AWLG13	500	500	500	—	—	—	—
EPLG5	—	—	—	250	—	—	500
Penicillin	—	—	—	4	4	0.125	0.125
Tetracycline	—	—	—	2	2	2	2
Nystatin	2	2	4	—	—	—	—
Cycloheximide	2	2	2	—	—	—	—

## Data Availability

The integer data used to support the findings of this study may be released upon application to the Institute of Biology, University of the Philippines. The Institute can be contacted at biology.upd@up.edu.ph.

## Supplementary Materials

Supplementary Materials Supplemental Figure 1: Optimization of the fermentation media for the antimicrobial activity using agar well assay. Zone of inhibition diameters of the supernatant of isolate AWLG9 grown in YMB against the yeast test microorganisms (a) *Candida albicans*, (b) *C. tropicalis*, and (c) *Saccharomyces cerevisiae.* Supplemental Figure 2: Optimization of the fermentation media for the antimicrobial activity using agar well assay. Zone of inhibition diameters of the supernatant of isolate AWLG8 grown in YMB against the yeast test microorganisms (a) *Candida albicans*, (b) *C. tropicalis*, and (c) *Saccharomyces cerevisiae.* Supplemental Figure 3: Optimization of the fermentation media for the antimicrobial activity using agar well assay. Zone of inhibition diameters of the supernatant of isolate AWLG10 grown in YMB against the yeast test microorganisms (a) *Candida albicans*, (b) *C. tropicalis*, and (c) *Saccharomyces cerevisiae*. Supplemental Figure 4: Optimization of the fermentation media for the antimicrobial activity using agar well assay. Zone of inhibition diameters of the supernatant of isolate AULG1 grown in YMB against the yeast test microorganisms (a) *Candida albicans*, (b) *C. tropicalis*, and (c) *Saccharomyces cerevisiae*. Supplemental Figure 5: Optimization of the fermentation media for the antimicrobial activity using agar well assay. Zone of inhibition diameters of the supernatant of isolate AWLG13 grown in YMB against the yeast test microorganisms (a) *Candida albicans*, (b) *C. tropicalis*, and (c) *Saccharomyces cerevisiae*. Supplemental Figure 6: Optimization of the fermentation media for the antimicrobial activity using agar well assay. Zone of inhibition diameters of the supernatant of isolate APLG2 grown in YMB against the yeast test microorganisms (a) *Candida albicans*, (b) *C. tropicalis*, and (c) *Saccharomyces cerevisiae*. Supplemental Figure 7: Optimization of the fermentation media for the antimicrobial activity of (a) AULG1 and (b) EPLG5 using agar well assay. Zone of inhibition diameters of the supernatant of isolates grown in YMB against the bacterial test microorganisms. Supplemental Figure 8: Optimization of the incubation time for the antimicrobial activity using agar well assay. Zone of inhibition diameters of the supernatant of isolate AWLG9 grown in YMB against the yeast test microorganisms (a) *Candida albicans*, (b) *C. tropicalis*, and (c) *Saccharomyces cerevisiae*. Supplemental Figure 9: Optimization of the incubation time for the antimicrobial activity using agar well assay. Zone of inhibition diameters of the supernatant of isolate AWLG8 grown in YMB against the yeast test microorganisms (a) *Candida albicans*, (b) *C. tropicalis*, and (c) *Saccharomyces cerevisiae*. Supplemental Figure 10: Optimization of the incubation time for the antimicrobial activity using agar well assay. Zone of inhibition diameters of the supernatant of isolate AWLG10 grown in YMB against the yeast test microorganisms (a) *Candida albicans*, (b) *C. tropicalis*, and (c) *Saccharomyces cerevisiae*. Supplemental Figure 11: Optimization of the incubation time for the antimicrobial activity using agar well assay. Zone of inhibition diameters of the supernatant of isolate AULG1 grown in YMB against the yeast test microorganisms (a) *Candida albicans*, (b) *C. tropicalis*, and (c) *Saccharomyces cerevisiae*. Supplemental Figure 12: Optimization of the incubation time for the antimicrobial activity using agar well assay. Zone of inhibition diameters of the supernatant of isolate AWLG13 grown in YMB against the yeast test microorganisms (a) *Candida albicans*, (b) *C. tropicalis*, and (c) *Saccharomyces cerevisiae*. Supplemental Figure 13: Optimization of the incubation time for the antimicrobial activity using agar well assay. Zone of inhibition diameters of the supernatant of isolate APLG2 grown in YMB against the yeast test microorganisms (a) *Candida albicans*, (b) *C. tropicalis*, and (c) *Saccharomyces cerevisiae*. Supplemental Figure 14: Optimization of the incubation time for the antimicrobial activity of (a) AULG1 and (b) EPLG5 using agar well assay. Zone of inhibition diameters of the supernatant of isolates grown in YMB against the bacterial test microorganisms.
