# Supplementary material for: Isolation of Actinomycetes with Cellulolytic and Antimicrobial Activities from Soils Collected from an Urban Green Space in the Philippines
Source: Int J Microbiol. 2021 Mar 17;2021:6699430. doi: 10.1155/2021/6699430 (PMC7990538; doi:10.1155/2021/6699430)
Supplement: Supplementary Materials — Supplemental Figure 1: Optimization of the fermentation media for the antimicrobial activity using agar well assay. Zone of inhibition diameters of the supernatant of isolate AWLG9 grown in YMB against the yeast test microorganisms (a) Candida albicans, (b) C. tropicalis, and (c) Saccharomyces cerevisiae. Supplemental Figure 2: Optimization of the fermentation media for the antimicrobial activity using agar well assay. Zone of inhibition diameters of the supernatant of isolate AWLG8 grown in YMB against the yeast test microorganisms (a) Candida albicans, (b) C. tropicalis, and (c) Saccharomyces cerevisiae. Supplemental Figure 3: Optimization of the fermentation media for the antimicrobial activity using agar well assay. Zone of inhibition diameters of the supernatant of isolate AWLG10 grown in YMB against the yeast test microorganisms (a) Candida albicans, (b) C. tropicalis, and (c) Saccharomyces cerevisiae. Supplemental Figure 4: Optimization of the fermentation media for the antimicrobial activity using agar well assay. Zone of inhibition diameters of the supernatant of isolate AULG1 grown in YMB against the yeast test microorganisms (a) Candida albicans, (b) C. tropicalis, and (c) Saccharomyces cerevisiae. Supplemental Figure 5: Optimization of the fermentation media for the antimicrobial activity using agar well assay. Zone of inhibition diameters of the supernatant of isolate AWLG13 grown in YMB against the yeast test microorganisms (a) Candida albicans, (b) C. tropicalis, and (c) Saccharomyces cerevisiae. Supplemental Figure 6: Optimization of the fermentation media for the antimicrobial activity using agar well assay. Zone of inhibition diameters of the supernatant of isolate APLG2 grown in YMB against the yeast test microorganisms (a) Candida albicans, (b) C. tropicalis, and (c) Saccharomyces cerevisiae. Supplemental Figure 7: Optimization of the fermentation media for the antimicrobial activity of (a) AULG1 and (b) EPLG5 using agar well assay. Zone of inhib [file 6699430.f1.pdf]

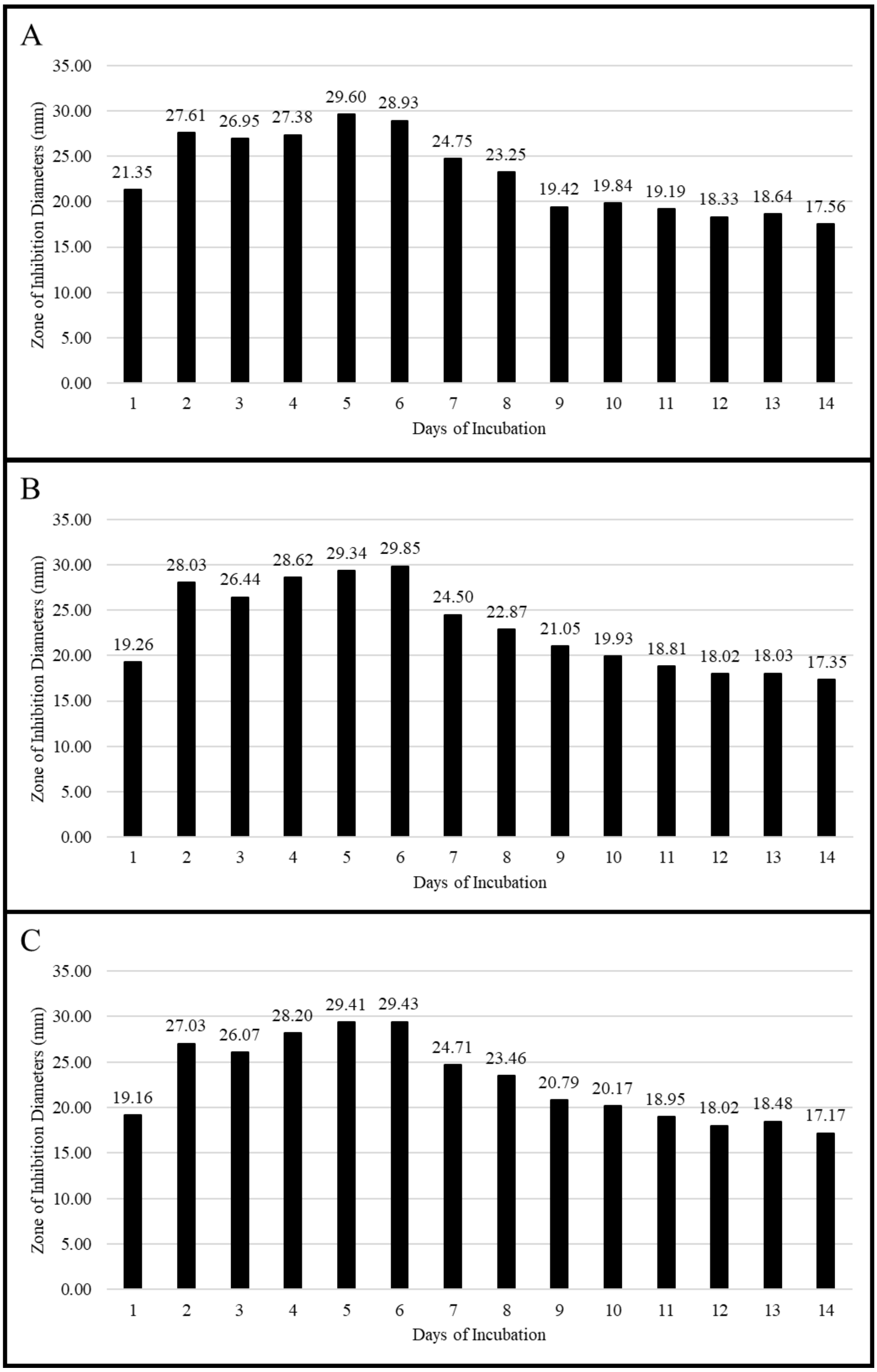

Supplemental Figure 1. Optimization of the fermentation media for the antimicrobial activity using agar well assay. Zone of inhibition diameters of the supernatant of isolate AWLG9 grown in YMB against the yeast test microorganism (A) *Candida albicans,* (B) *C. tropicalis,* and (C). *Saccharomyces cerevisiae.*

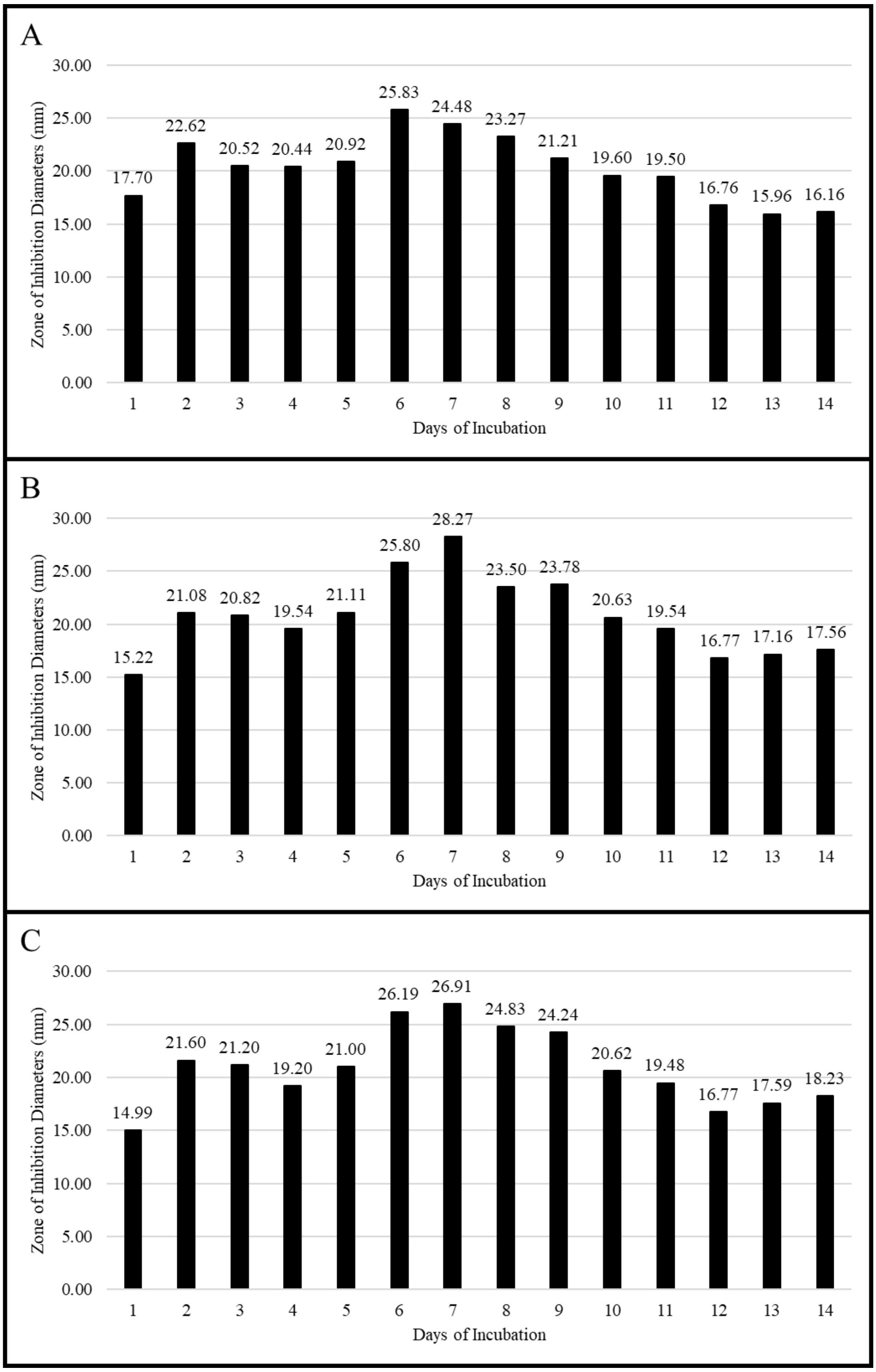

Supplemental Figure 2. Optimization of the fermentation media for the antimicrobial activity using agar well assay. Zone of inhibition diameters of the supernatant of isolate AWLG8 grown in YMB against the yeast test microorganism (A) *Candida albicans,* (B) *C. tropicalis,* and (C). *Saccharomyces cerevisiae.*

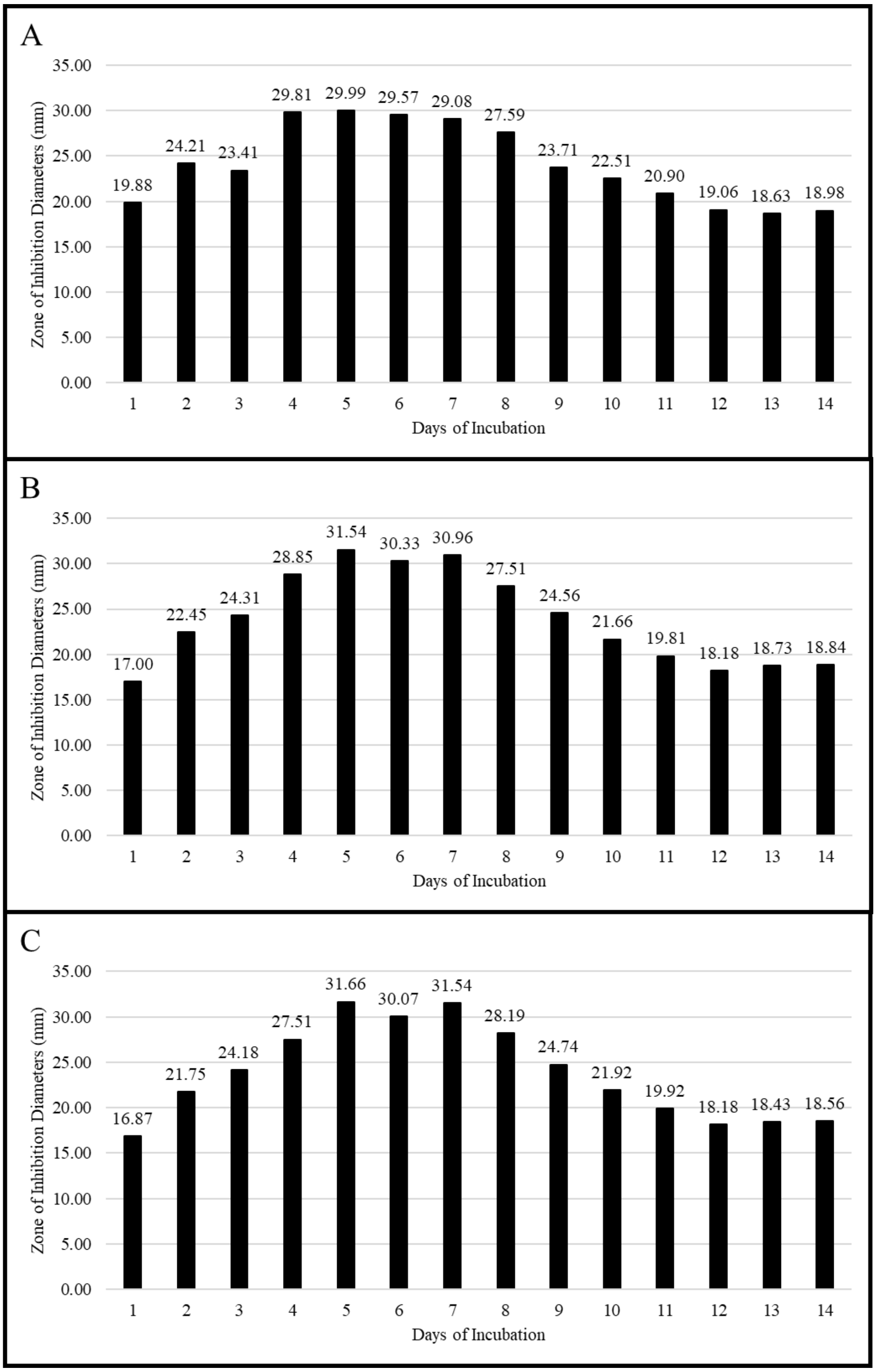

Supplemental Figure 3. Optimization of the fermentation media for the antimicrobial activity using agar well assay. Zone of inhibition diameters of the supernatant of isolate AWLG10 grown in YMB against the yeast test microorganism (A) *Candida albicans,* (B) *C. tropicalis,* and (C). *Saccharomyces cerevisiae.*

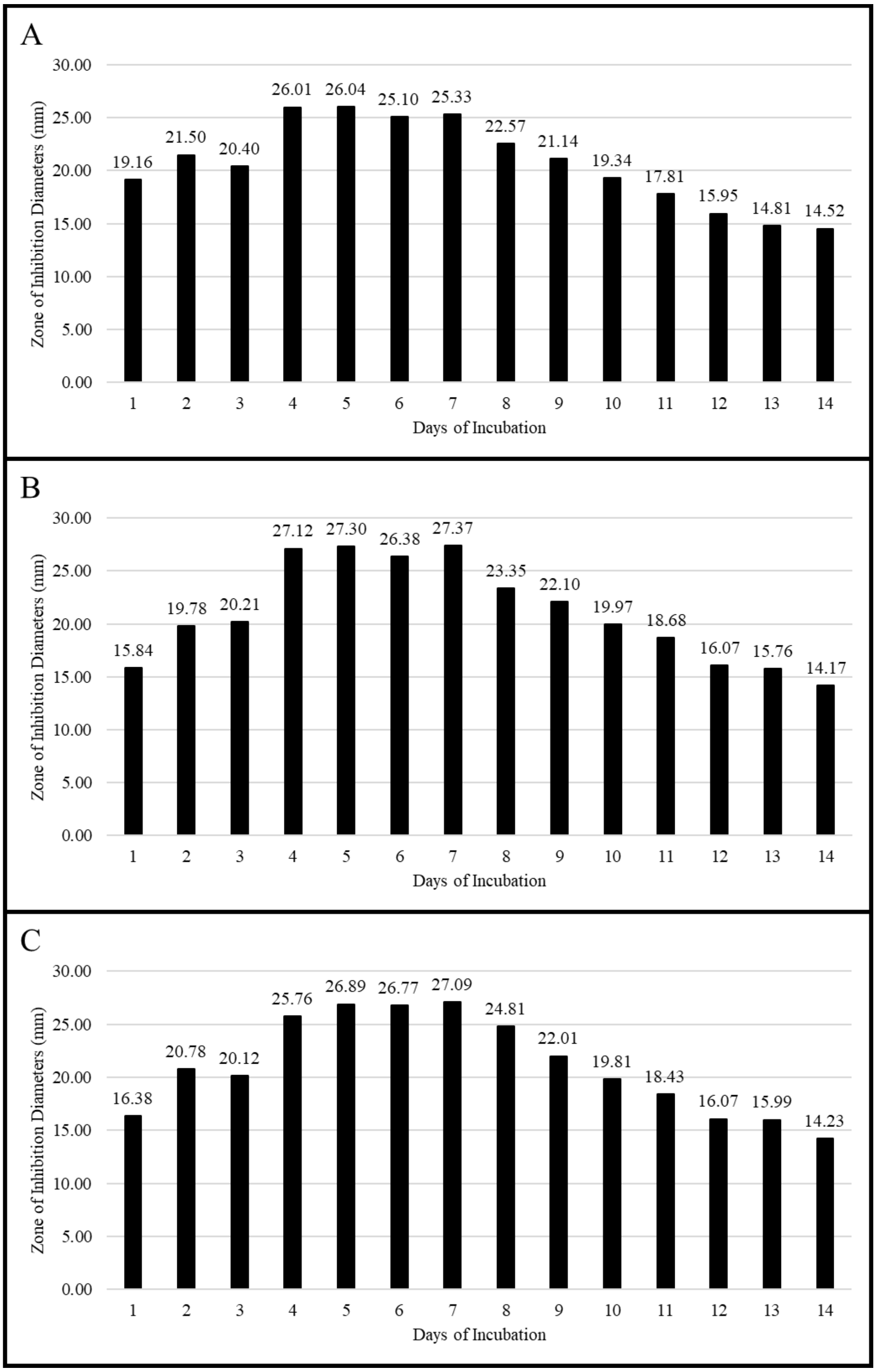

Supplemental Figure 4. Optimization of the fermentation media for the antimicrobial activity using agar well assay. Zone of inhibition diameters of the supernatant of isolate AULG1 grown in YMB against the yeast test microorganism (A) *Candida albicans,* (B) *C. tropicalis,* and (C). *Saccharomyces cerevisiae.*

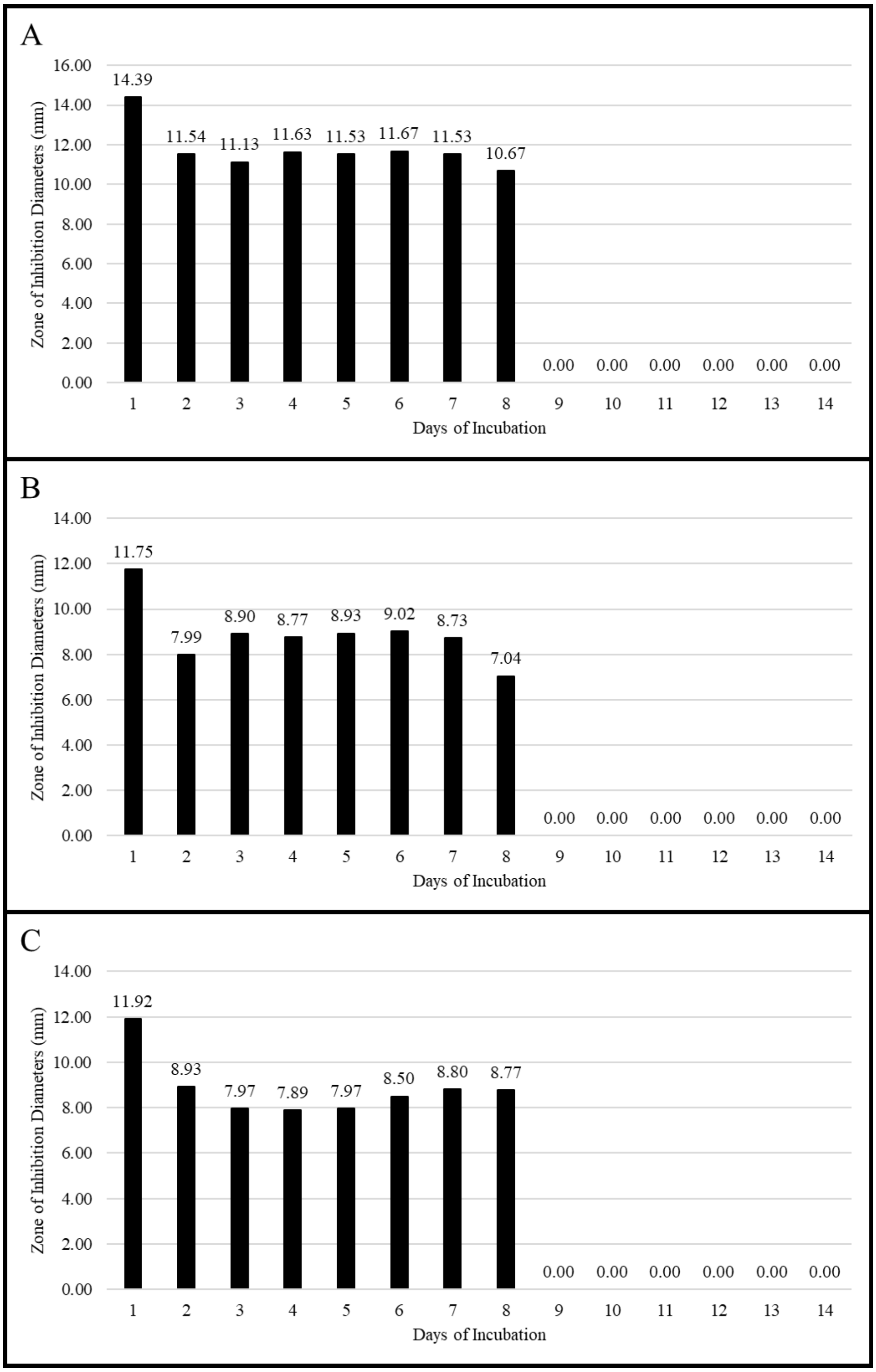

Supplemental Figure 5. Optimization of the fermentation media for the antimicrobial activity using agar well assay. Zone of inhibition diameters of the supernatant of isolate AWLG13 grown in YMB against the yeast test microorganism (A) *Candida albicans,* (B) *C. tropicalis,* and (C). *Saccharomyces cerevisiae.*

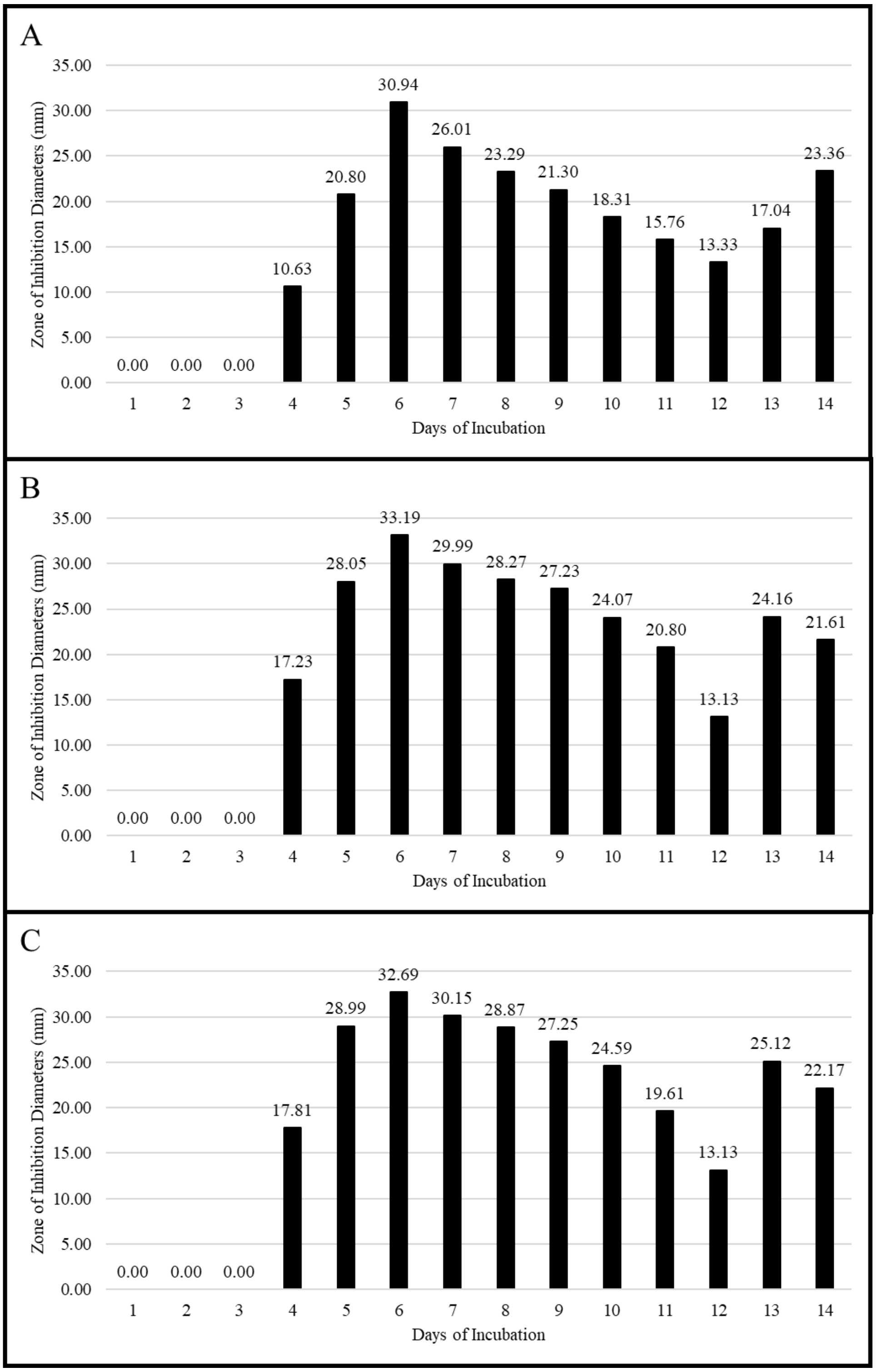

Supplemental Figure 6. Optimization of the fermentation media for the antimicrobial activity using agar well assay. Zone of inhibition diameters of the supernatant of isolate APLG2 grown in YMB against the yeast test microorganism (A) *Candida albicans,* (B) *C. tropicalis,* and (C). *Saccharomyces cerevisiae.*

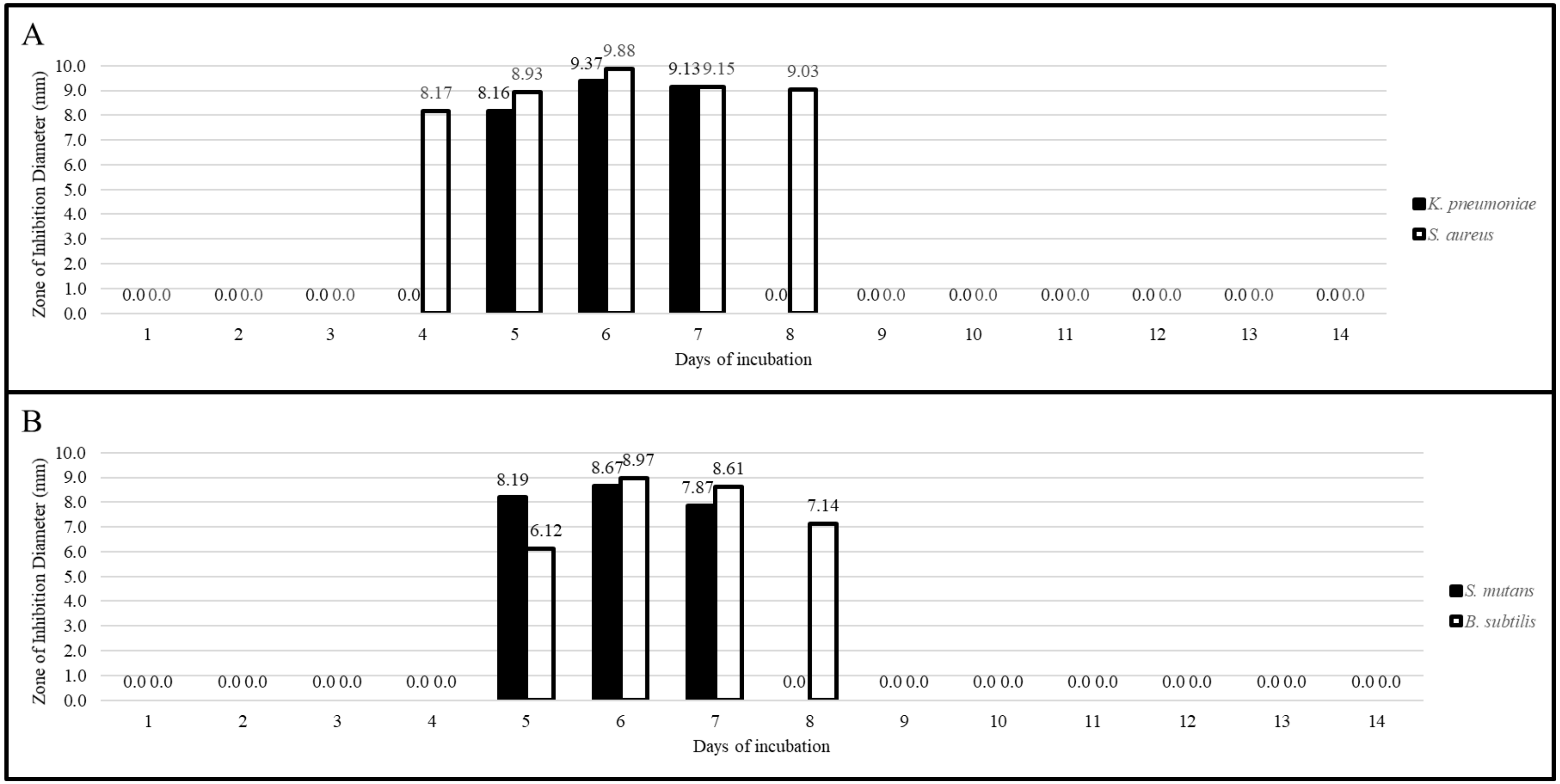

Supplemental Figure 7. Optimization of the fermentation media for the antimicrobial activity of (A) AULG1 and (B) EPLG5 using agar well assay. Zone of inhibition diameters of the supernatant of isolates grown in YMB against the bacterial test microorganisms.

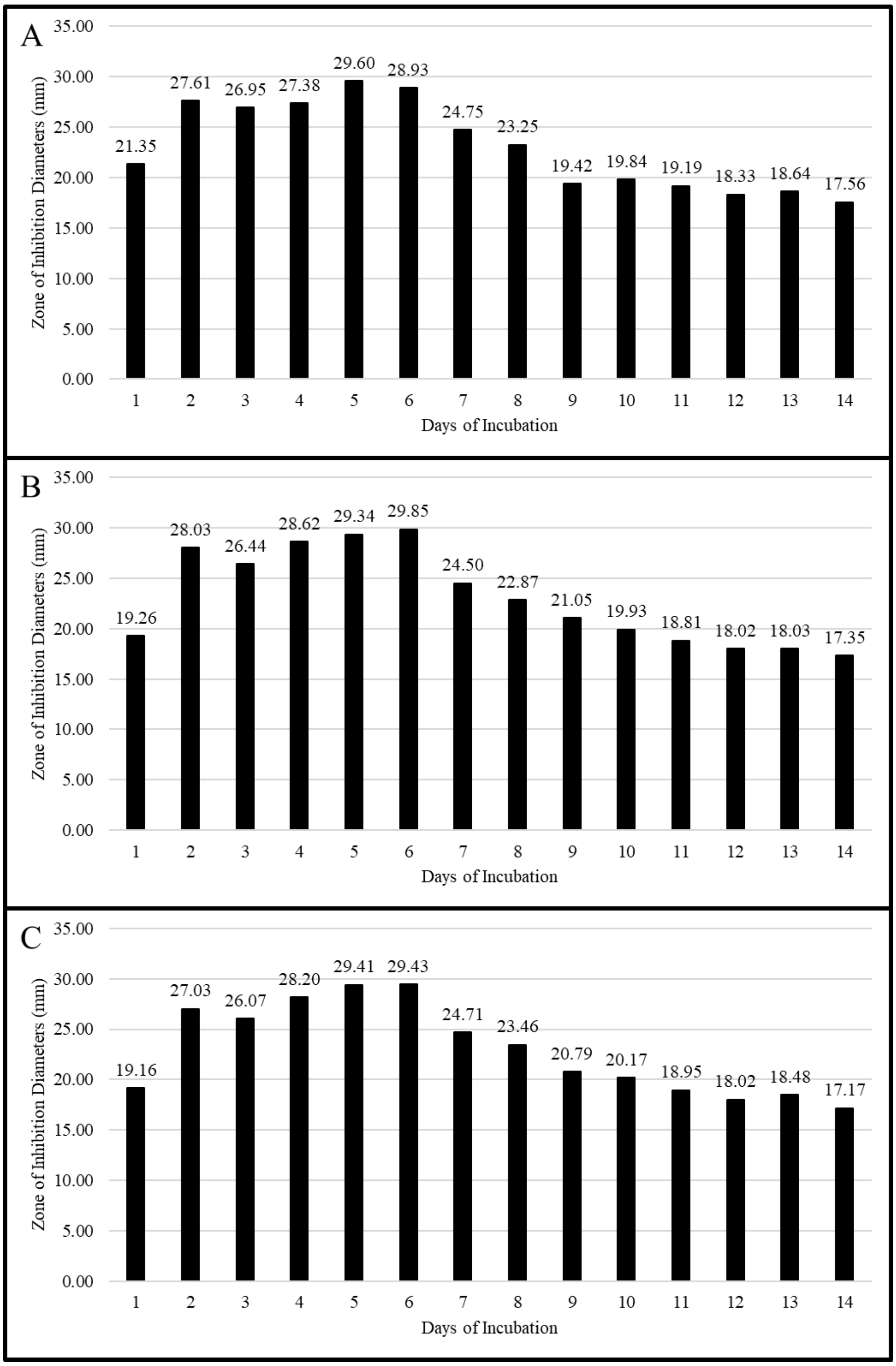

Supplemental Figure 8. Optimization of the incubation time for the antimicrobial activity using agar well assay. Zone of inhibition diameters of the supernatant of isolate AWLG9 grown in YMB against the yeast test microorganism (A) *Candida albicans,* (B) *C. tropicalis,* and (C). *Saccharomyces cerevisiae.*

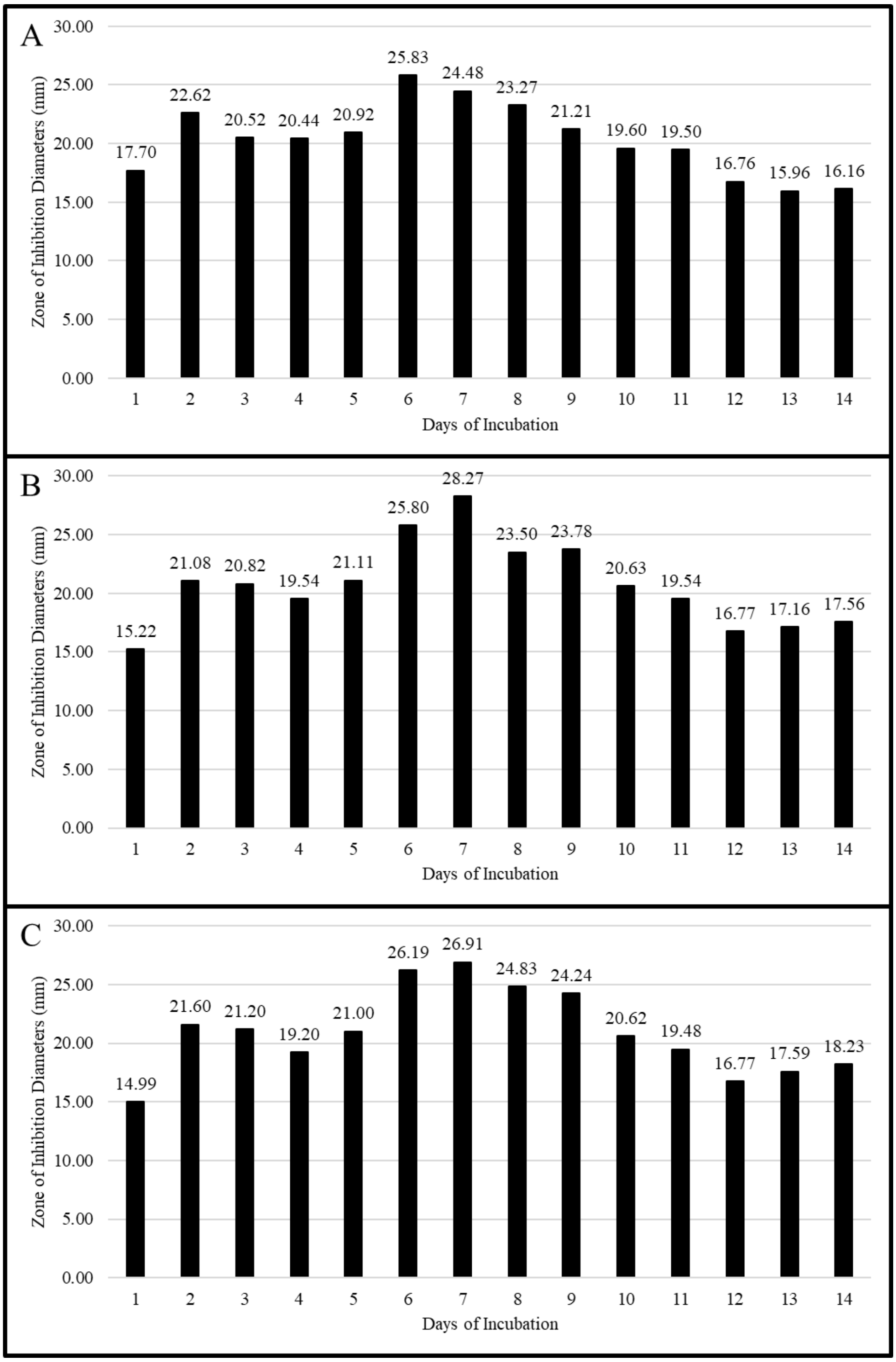

Supplemental Figure 9. Optimization of the incubation time for the antimicrobial activity using agar well assay. Zone of inhibition diameters of the supernatant of isolate AWLG8 grown in YMB against the yeast test microorganism (A) *Candida albicans,* (B) *C. tropicalis,* and (C). *Saccharomyces cerevisiae.*

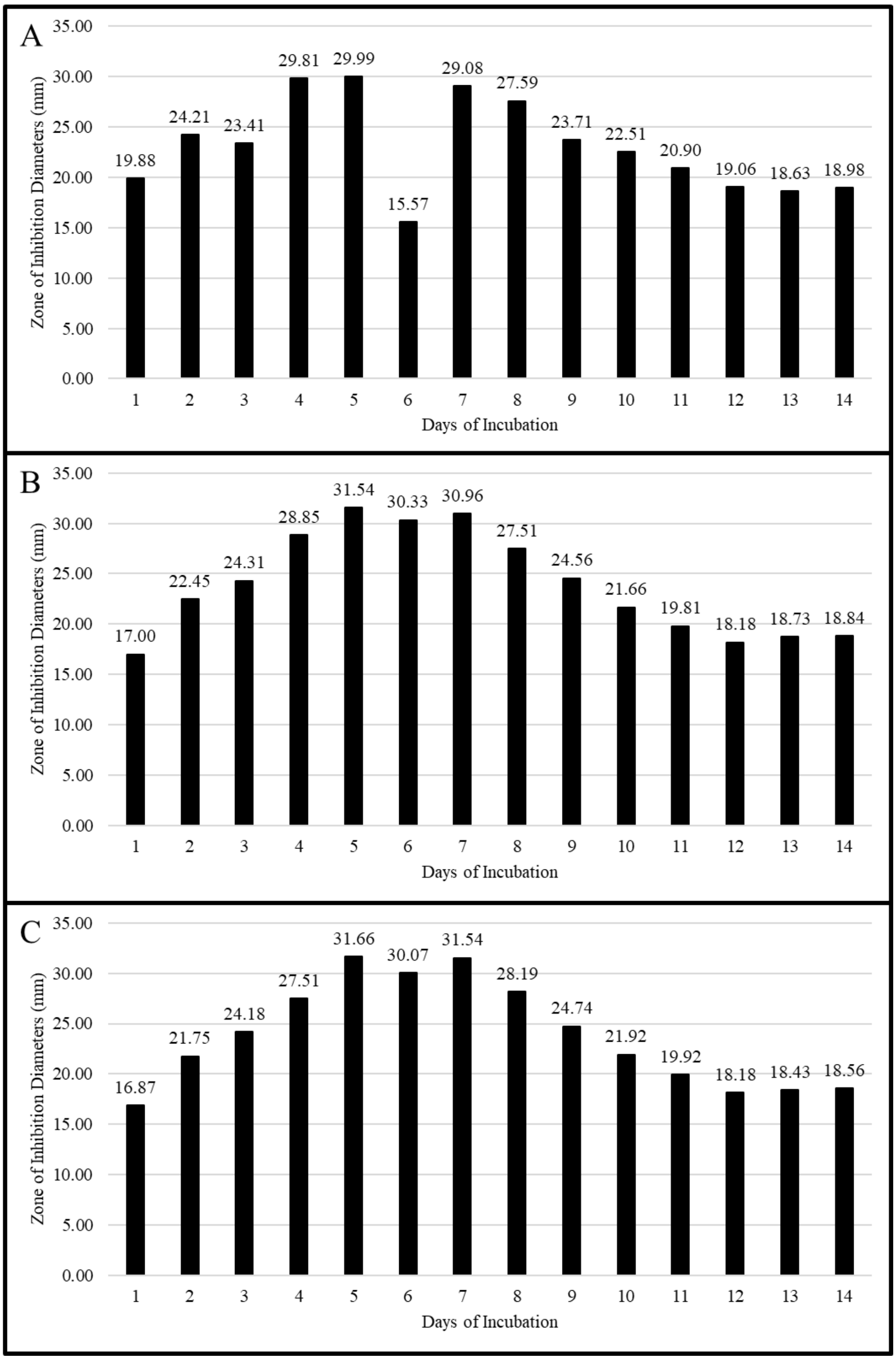

Supplemental Figure 10. Optimization of the incubation time for the antimicrobial activity using agar well assay. Zone of inhibition diameters of the supernatant of isolate AWLG10 grown in YMB against the yeast test microorganism (A) *Candida albicans,* (B) *C. tropicalis,* and (C). *Saccharomyces cerevisiae.*

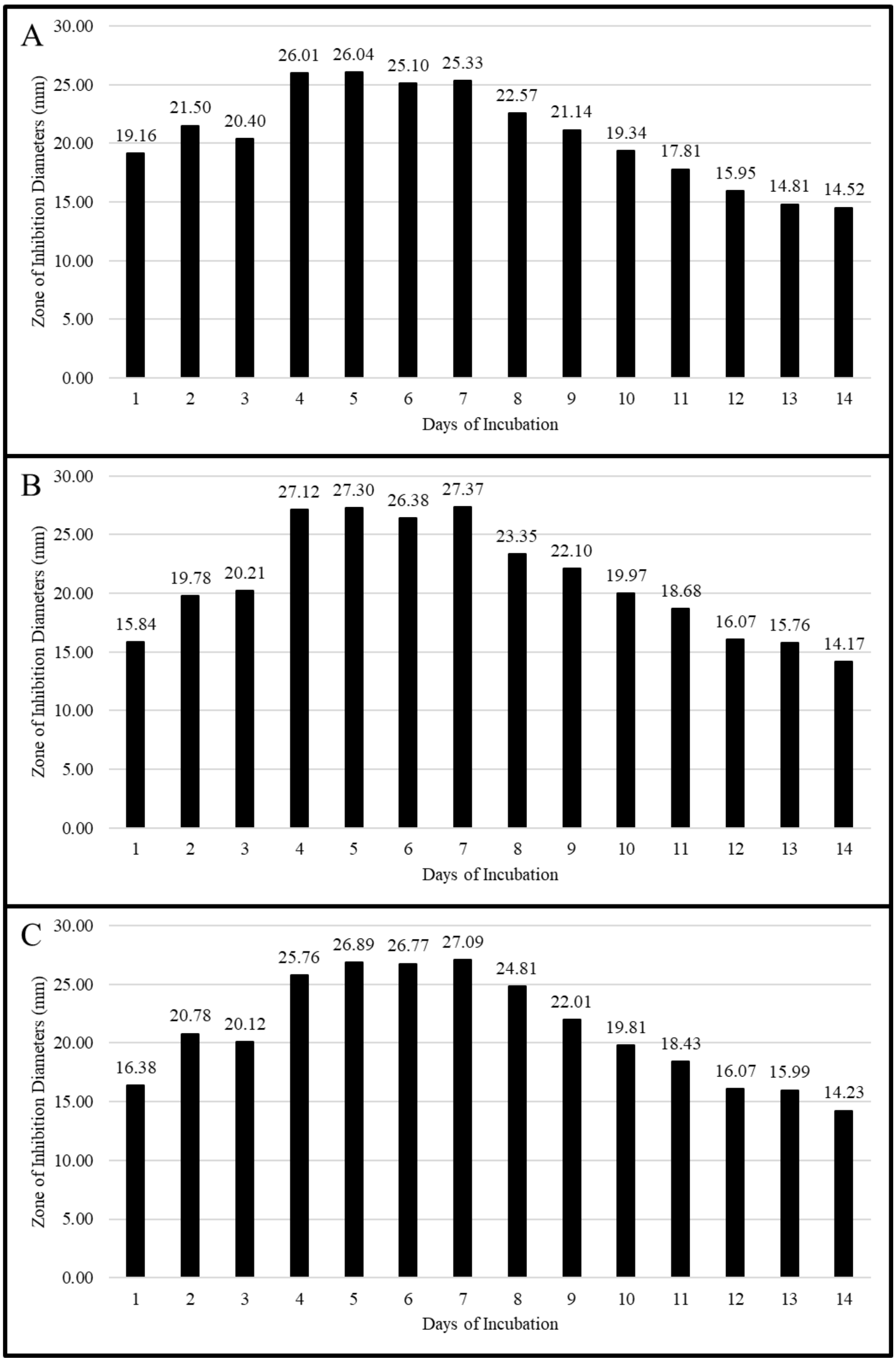

Supplemental Figure 11. Optimization of the incubation time for the antimicrobial activity using agar well assay. Zone of inhibition diameters of the supernatant of isolate AULG1 grown in YMB against the yeast test microorganism (A) *Candida albicans,* (B) *C. tropicalis,* and (C). *Saccharomyces cerevisiae.*

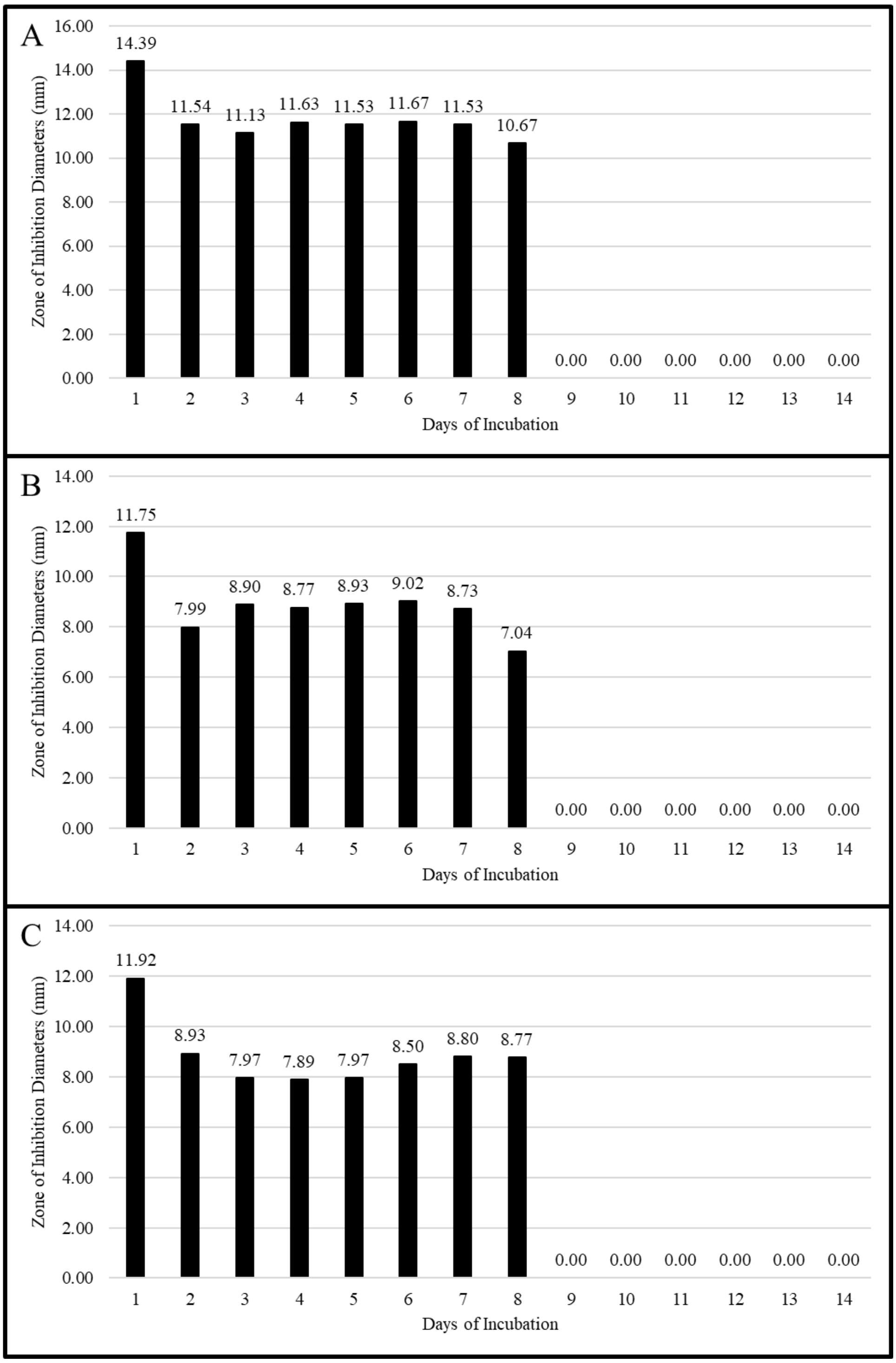

Supplemental Figure 12. Optimization of the incubation time for the antimicrobial activity using agar well assay. Zone of inhibition diameters of the supernatant of isolate AWLG13 grown in YMB against the yeast test microorganism (A) *Candida albicans,* (B) *C. tropicalis,* and (C). *Saccharomyces cerevisiae.*

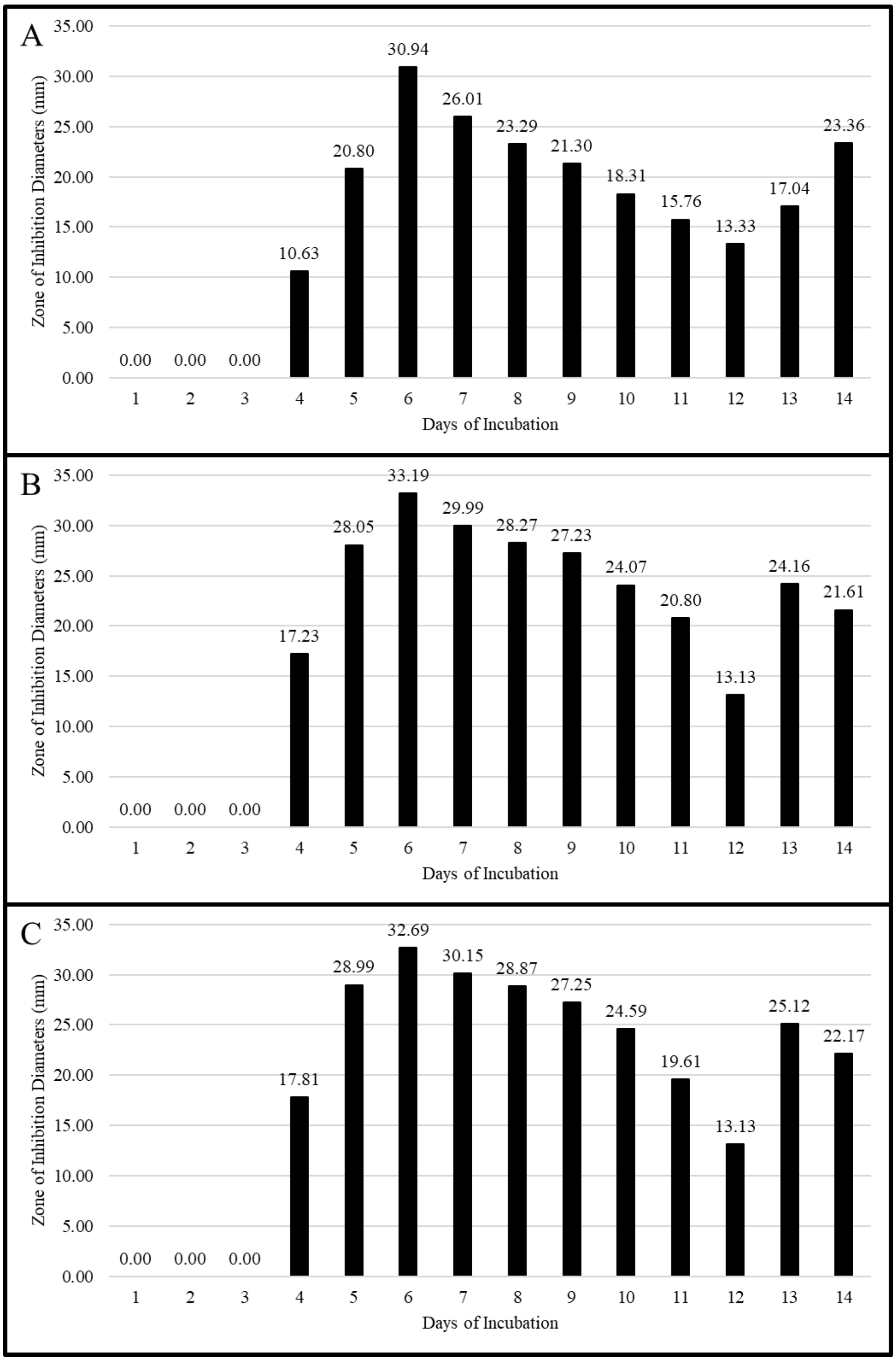

Supplemental Figure 13. Optimization of the incubation time for the antimicrobial activity using agar well assay. Zone of inhibition diameters of the supernatant of isolate APLG2 grown in YMB against the yeast test microorganism (A) *Candida albicans,* (B) *C. tropicalis,* and (C). *Saccharomyces cerevisiae.*

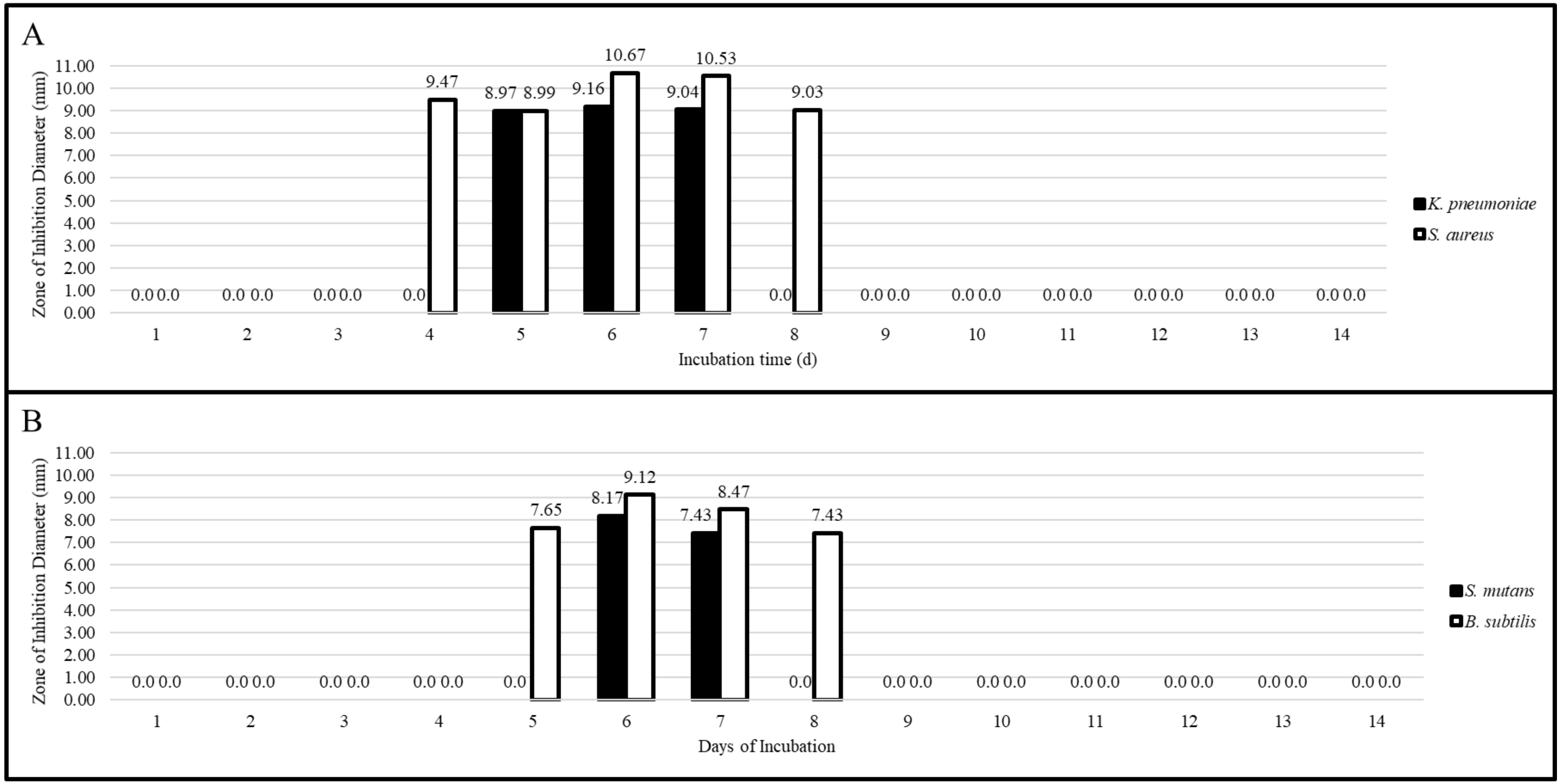

Supplemental Figure 14. Optimization of the incubation time for the antimicrobial activity of (A) AULG1 and (B) EPLG5 using agar well assay. Zone of inhibition diameters of the supernatant of isolates grown in YMB against the bacterial test microorganisms.
